# Palliative care training in medical undergraduate education: a survey among the faculty

**DOI:** 10.1186/s12904-024-01351-4

**Published:** 2024-01-18

**Authors:** Kadri Suija, Stephen R. Mason, Frank Elsner, Piret Paal

**Affiliations:** 1 Institute of Family Medicine and Public Health, Faculty of Medicine, University of Tartu, Tartu, Estonia; 2 Institute of Public Health and Clinical Nutrition, Faculty of Health Sciences, University of Eastern Finland, Kuopio, Finland; 3 Palliative Care Unit, Health and Life Sciences, Life Course and Medical Sciences, University of Liverpool, Room G036, 200 London Road, Liverpool, L3 9TA UK; 4 Department of Palliative Medicine, Medical Faculty, RWTH Aachen University, Aachen, Germany; 5 Institute of Palliative Care, Paracelsus Medical University, Salzburg, Austria

**Keywords:** Palliative care, Medical student education, Educational design

## Abstract

**Background:**

A minority of European countries have compulsory training in palliative care within all medical schools. The aim of the study was to examine palliative care education in Estonia.

**Methods:**

We used the adapted version of the Palliative Education Assessment Tool (PEAT) to evaluate palliative care education at the University of Tartu, the only medical school in Estonia. The PEAT comprises of different palliative care domains and allows for assessing the curricula for palliative care education.

**Results:**

26 hours (h) of palliative care is taught within the basic medical curriculum, which is divided between 14 courses. Ethical issues (4 h, lecture and seminar) and basics of palliative care (2.5 h, lecture) are well covered however, pain and symptom management (12.5 h, lecture, seminar, workshop), psychosocial, spiritual aspects (5.5 h, seminar), and communication (1.5 h, lecture) teaching do not reach the recommended number of hours. Teamwork and self-reflection are not taught at all.

**Conclusions:**

Increased time, more diverse teaching strategies and clear learning outcomes are required to enable the development of palliative care education in Estonia. The teaching and learning of palliative care is a process that requires constant development and collaboration.

**Supplementary Information:**

The online version contains supplementary material available at 10.1186/s12904-024-01351-4.

## Background

The changing demographic profile of the European population highlights an increasingly aged population, illustrating the growing need for palliative care [1]. Moreover, providing high-quality palliative care training for undergraduate medical students is one of the main keys to safeguarding access to palliative care [2, 3].

In 2019, the Atlas of Palliative Care in Europe reported that only nine countries in Europe have compulsory training in palliative care in all medical schools [4]. One of these countries is Estonia. With just over 1.3 million inhabitants, Estonia is one of the least populous members of the European Union. In Estonia there is only one medical school; the Faculty of Medicine at the University of Tartu, founded in 1636.

In 2019, the European Association for Palliative Care (EAPC) published updated recommendations for palliative care curriculum development, which includes seven main topics: (1) basics of palliative care, (2) psychosocial and spiritual issues, (3) pain management, (4) symptom control, (5) ethical and legal issues, (6) communication, and (7) teamwork and self-reflection [5]. Based on the experience of Romania, one of the first countries to implement these recommendations, Medical Schools had to allocate time to teach palliative care, and faculty competencies and skills needed updating [6].

In Finland, recommendations for palliative care education were formulated by a nationally funded programme. The final report identified ways to harmonise teaching, for example by formulating highly relevant topics. The report also noted that basic knowledge and skills must be covered in a separate/distinct course, while the additional expertise can be taught through ‘horizontal integration’ within different courses [7].

Several studies indicated that it is important to understand the current state of teaching of palliative care, and to examine it systematically and regularly [8, 9]. Although Estonia is highly ranked in the EAPC Atlas for having mandatory palliative care training, the state of palliative care teaching and learning has not been specifically studied. The aim of the study was to examine palliative care education in Estonia.

## Methods

To understand the current state of palliative care education and training in Estonia, we conducted a survey among the faculty at the University of Tartu. The Palliative Education Assessment Tool (PEAT) was obtained as a survey inventory [10]. The PEAT consists of seven areas, each with specific curricular objectives, such as: (1) palliative care; (2) pain; (3) neuropsychological symptoms; (4) other symptoms, (5) ethics and law, (6) patient/family and non-clinical caregiver perspectives on end-of-life care, and (7) clinical communication skills. It has been designed to help inform the development of curricula for palliative care education [10].

First, we translated and adapted the PEAT. We used forward-backward-translation method, using the principles outlined in the translation guideline [11] - which means that the instrument was translated into Estonian by one translator and then back to English by another translator. No significant differences in the translations were found. Later we performed the instrument pilot testing. We decided to exclude the ‘neuropsychological symptoms’ and added the domain of ‘specific issues about end-of-life care‘. The original domain focusses on neuropsychological symptoms associated with dying, including those that are related to medications. As there may be various symptoms associated with dying, and side-effects of medications are covered in symptom care, we decided that ‘specific issues about end-of-life care‘ would be a more meaningful domain.

Secondly, we prepared the questions in table format and tested the acuity of the questions by entering data from two courses that cover aspects of palliative care. We used the data available in the learning database of the University. We pilot-tested the PEAT with faculty members (*n* = 5) involved in teaching palliative care (the PEAT is available as a supplementary file).

In January 2023, we emailed the survey to all heads of departments of the faculty of medicine (*n* = 23). We asked if they teach palliative care in their discipline to medical students and if yes, to detail content and teaching time. We gave three weeks to complete the survey, and after a reminder granted an additional week. In February, we contacted all clinical teachers who responded to acquire further information about teaching methods and performance assessment, which was not found in the learning database.

All methods were carried out in accordance with relevant local regulations. The Ethics Committee of the University of Tartu was contacted but waived ethical approval and a need for informed consent as the information collected and analysed was available in the learning database of the University of Tartu, which is freely available. In addition, the analysis was considered as a service evaluation. No personal data was collected.

## Results

Of 23 persons contacted, 13 responded and seven reported about teaching palliative medicine in their discipline. Data received provided information on 14 courses covering various aspects of palliative care. Of these, ten courses were for undergraduate medical students, two for dentistry students, one within the physiotherapy curriculum, and one for post-graduate studies (medical residents). Within the medical curriculum, seven courses were compulsory for all students, and three were elective. The participants who reported that they were teaching palliative medicine within basic medical curriculum were from the following disciplines: medical theory and ethics, geriatrics, clinical pharmacology, pulmonology and thoracic surgery, family medicine, neurology and neurosurgery, rehabilitation medicine (Table 1).

**Table 1:** Palliative care teaching in the Faculty of Medicine, University of Tartu

Year	Speciality Course	Palliative care topics and aims	Teaching method	Performance assessment	Palliative care teaching hours
I	Medical Theory and Ethics	Ethics at the end of life	Lecture, seminar	Written examination, where there are also questions about end-of-life care ethics	4 h (2 h lecture and 2 h seminar)
IV	Geriatrics	Principles of palliative care, Communication and End-of-life care with a focus on older people	Seminar	Multiple-choice test, where there are questions also about elderly palliative care	3 h
IV	Clinical Pharmacology	Pharmacological treatment of pain	Seminar	None, but participation in the seminar is obligatory	4 h
IV	Pulmonology and Thoracic Surgery	Management of the chronic progressive lung disease, fibrotic interstitial lung disease, pulmonary hypertension	Lecture	None but participation is obligatory	0.5 h
V	Family Medicine II	General principles of palliative care, symptom assessment, pain care, nausea, constipation, dyspnoea management, general principles of end-of-life care, teamwork and communication with a focus on primary care	Lecture, workshop	One clinical case about primary palliative care in the written examination	5 h (2 h lecture and 3 h workshop)
V	Neurology and Neurosurgery	Management of the patients with neurodegenerative diseases	Seminar	Oral examination, where there are also questions about palliative care of neurological patients	7.5 h
V	Rehabilitation Medicine	Nutrition and nutrition-related problems management, oncological rehabilitation	Seminar	None, but participation in the seminar is obligatory	2 h

The dentistry curriculum covers aspects including end-of-life care ethics (4 h (h)) and dental care of the elderly and chronically ill people (6 h). The physiotherapy curriculum covers aspects of end-of-life care ethics (2 h). There is one theoretical course (26 h) for post-graduate medical students, which covers topics such as the general principles of palliative care (4 h), pain and other symptom care (12 h), end-of-life care (1 h), ethical and legal issues (4 h), communication (3 h), and supporting relatives (2 h).

There are also elective courses and medical students may meet palliative patients during their practical training in hospitals and primary care centres, but the elective courses do not take place every year and not all students take them. The opportunities for practical clinical training also vary, and therefore, we have not included elective courses in this analysis.

### Comparisons with the EAPC recommended curriculum

**Fig. 1:**
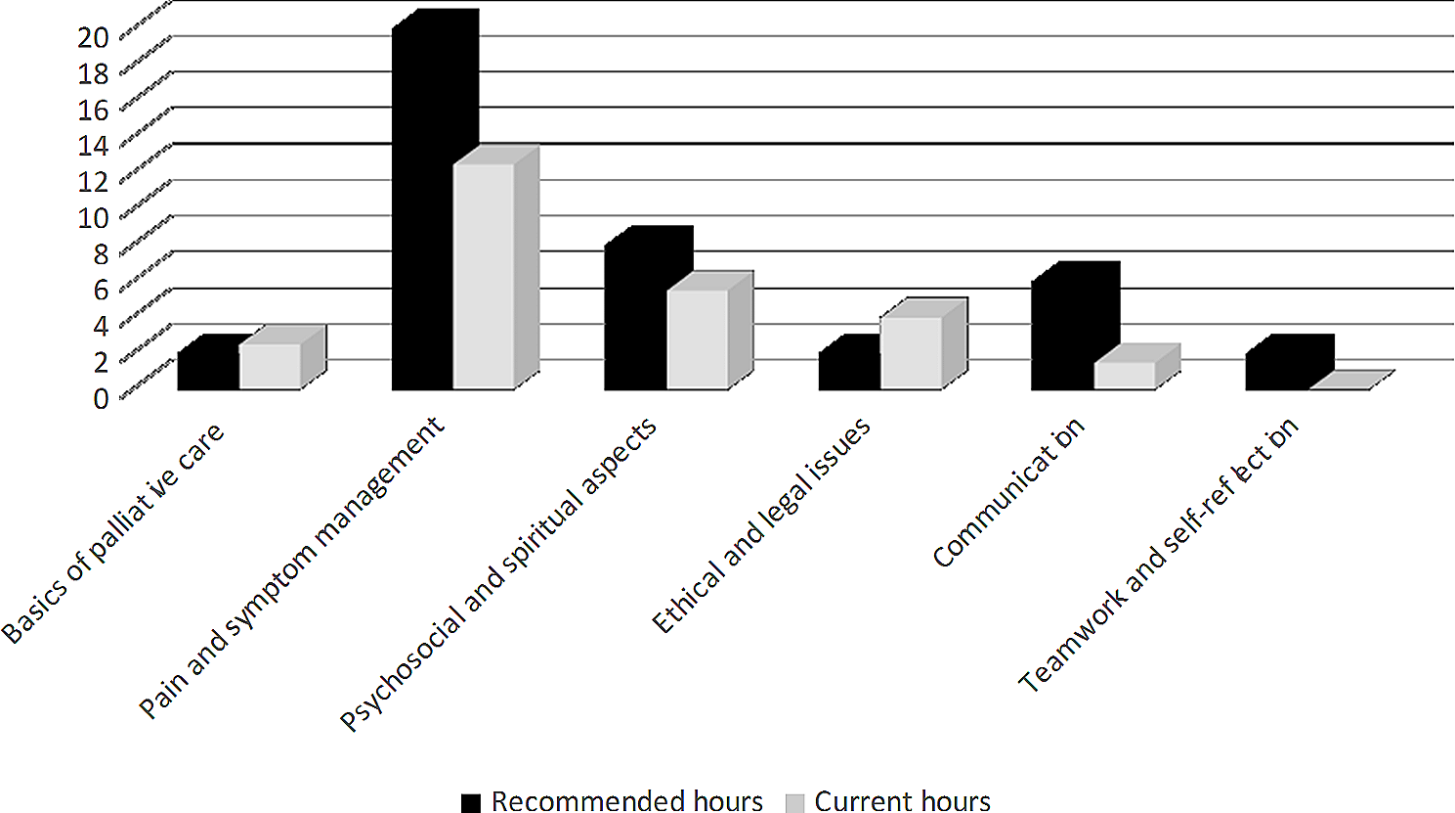
EAPC recommended hours and current hours of palliative care in the medical curriculum

26 h of palliative care are taught as part of the basic medical curriculum (total number of points according to the European Credit Transfer and Accumulation System is 360) (see Table 1). Comparing the palliative care topics covered in the undergraduate medical curriculum with the EAPC recommendations identifies that the general principles of palliative care (2.5 h, lecture based) are well covered, however, pain and symptom management (12.5 h, lecture, seminar, workshop), and psychosocial and spiritual aspects (5.5 h, seminar based) do not reach the recommended number of hours. Communication training (1.5 h, lecture based) needs more allocated time to match EAPC recommendations (15% of training time). The ethical and legal aspects of palliative care at the University of Tartu are 4 h (lecture and seminar) and exceed EAPC recommendations (5%/up to 2 h). Teamwork and self-reflection are not currently taught. Findings are summarised in Fig. 1.

## Discussion

This first report on the provision of palliative care education in Estonia demonstrates that although highly ranked in the EAPC Atlas, several issues in the palliative care curriculum need revision and improvement. The 26 h of mostly lecture or seminar-based teaching across various disciplines in first, fourth and fifth year is used for palliative care education within the basic medical curriculum in Estonia. The learning goals as well as the performance assessment varies across the courses. This means that a significant collaboration and planning is necessary to ensure meaningful training.

These results accord with information from systematic reviews of palliative care education, which report that teaching tends to be fragmented and uncoordinated or concentrates more on knowledge acquisition, rather than skills and attitudes. In addition, palliative care education is rarely formally assessed, suggesting that the effectiveness of training is not considered and the context for revision and improvements is limited. Further, there are difficulties in recruiting suitable teachers [12, 13].

When comparing the training in Estonia with the recommendations of the Finnish report, it is noticeable that horizontal integration (teaching within different speciality courses) is used more in Estonia than in Finland. This may improve the integration of palliative care, but care needs to be taken that there is parity in horizontal integration within speciality courses [7].

The main teaching methods in Estonia were lectures and seminars, which when appropriately structure can effectively disseminate knowledge and provide opportunities for critical discussion and reflection with a large number of students. Less were used workshops, and there was no examples of bedside training, simulation, or specific skills teaching. There is strong evidence that improved self-efficacy and outcome expectancies result in behavioural changes in medical students that lead to improved practice and better patient care [14]. Such changes are facilitated by sufficient time for training, and more importantly, engaging appropriate content and teaching strategies. There is no ‘best way’ to teach palliative care, although studies have shown that practice-based teaching, supported by experiential exercises and using appropriate assessment techniques is optimal [13]. The lack of practical, for example bedside learning, as well as overarching learning goals and suitable performance assessment are shortcomings in teaching. There are few practical training places for palliative care available, and not enough qualified tutor doctors/teachers in Estonia. Preparing such training needs commitment and skills, and requires investment at the university level to improve educators’ knowledge about immersive training methods. The importance of collaborative planning to develop overarching learning objectives for all courses was reported by Afshar et al., in their study about the development of the interdisciplinary and cross-sectional palliative care course at the Hannover Medical School [15].

It is clear that if there is no overall assessment which is related to learning goals and teaching methods, we cannot know how well newly qualified doctors are prepared for palliative care. A recent systematic review identified that palliative care education has a significant positive effect on junior doctors’ emotional well-being and professional attitudes. Further, around half of the medical students felt that their medical education is failing to prepare them to deliver good end-of-life care, and most students did not feel prepared to address ethical challenges at the end-of-life [16]. To change this, palliative care professors across Europe believe that a general increase in awareness of society, including medical students, of the importance of dealing properly with death, dying, and suffering is needed, and therefore, palliative care should be appropriately integrated into all education [6]. Furthermore, while general recommendations such as the EAPC recommendation for basic medical education are helpful, each country has its own problems and medical education curricula need to be adapted to local conditions [8, 13, 15, 17].

This study highlights the need for future research to assess the differential impact of small and large educational interventions in palliative care, whether interventions lead to behaviour change, and how teaching affects clinical practice. The impact of teaching on specific elements of patient care also needs to be explored and could utilise markers of clinical assessment, management and patient/family feedback.

### Strength and limitations

This is the first time that palliative care education has been mapped in Estonia. Data were collected directly from educators, so may be open to over or under-representation/application using the PEAT [10]. No information was collected from students in this study, and this should be engaged in future studies.

As curricula in medicine are usually already full, it can be difficult for new specialities such as palliative medicine, to find and maintain their place and teaching points. Finding optimal ways to integrate palliative care into existing curricula necessitates regular assessments to enable meaningful review. We hope that results of this study will support not only development of palliative care education but also medical curricula in general. The results from countries where the development of palliative care is still ongoing, such as Estonia, are valuable for other countries and may help to identify the most important deficits and where to make improvements. More harmonization in palliative care education is needed.

## Conclusions

Increased time, more diverse teaching strategies and clear learning outcomes are required to enable the development of palliative care education in Estonia. The teaching and learning of palliative care is a process that requires constant development and collaboration.

### Electronic supplementary material

Below is the link to the electronic supplementary material.

**Supplementary Material 1:** The adapted version of the Palliative Education Assessment Tool (PEAT), which was used as the survey inventory

## Data Availability

All data generated or analysed during this study are included in this published article.

## Abbreviations

EAPC: The European Association for Palliative Care
PEAT: The Palliative Education Assessment Tool
hours: h
