# Supplementary material for: Palliative care training in medical undergraduate education: a survey among the faculty
Source: BMC Palliat Care. 2024 Jan 18;23:19. doi: 10.1186/s12904-024-01351-4 (PMC10795347; doi:10.1186/s12904-024-01351-4)
Supplement: Supplementary file 1 — Supplementary Material 1: The adapted version of the Palliative Education Assessment Tool (PEAT), which was used as the survey inventory [file 12904_2024_1351_MOESM1_ESM.pdf]

Supplementary Table. The adapted version of the Palliative Education Assessment Tool (PEAT), which was used as the survey inventory

Domain/topic	Included in the programme yes/no	Time (in academic hours)	Name of the course	The course code in the learning database	Content of the course (including teaching methods, performance assessment)
Palliative medicine: basic concepts and principles					
Pain management					
Management of other symptoms, e.g. dyspnoe, nausea, constipation, delirium, etc.					
End-of-life care issues					
Ethics and legal issues surrounding					

end of life					
Patient/family/ caregived non-clinical perspective					
Communication skills specific to end of life					
